# Population pharmacokinetics of voriconazole and initial dosage optimization in patients with talaromycosis

**DOI:** 10.3389/fphar.2022.982981

**Published:** 2022-09-26

**Authors:** Zhiwen Jiang, Yinyi Wei, Weie Huang, Bingkun Li, Siru Zhou, Liuwei Liao, Tiantian Li, Tianwei Liang, Xiaoshu Yu, Xiuying Li, Changjing Zhou, Cunwei Cao, TaoTao Liu

**Affiliations:** ^1^ Department of Dermatology and Venereology, The First Affiliated Hospital of Guangxi Medical University, Nanning, China; ^2^ Guangxi Health Commission Key Lab of Fungi and Mycosis Research and Prevention, Nanning, China; ^3^ Department of Pharmacy, The First Affiliated Hospital of Guangxi Medical University, Nanning, China; ^4^ Department of Infectious Diseases, Baise People’s Hospital, Baise, China

**Keywords:** voriconazole, talaromycosis, population pharmacokinetics, dosage optimization, Monte Carlo simulation, C- reactive protein (CRP)

## Abstract

The high variability and unpredictability of the plasma concentration of voriconazole (VRC) pose a major challenge for clinical administration. The aim of this study was to develop a population pharmacokinetics (PPK) model of VRC and identify the factors influencing VRC PPK in patients with talaromycosis. Medical records and VRC medication history of patients with talaromycosis who were treated with VRC as initial therapy were collected. A total of 233 blood samples from 69 patients were included in the study. A PPK model was developed using the nonlinear mixed-effects models (NONMEM). Monte Carlo simulation was applied to optimize the initial dosage regimens with a therapeutic range of 1.0–5.5 mg/L as the target plasma trough concentration. A one-compartment model with first-order absorption and elimination adequately described the data. The typical voriconazole clearance was 4.34 L/h, the volume of distribution was 97.4 L, the absorption rate constant was set at 1.1 h^-1^, and the bioavailability was 95.1%. Clearance was found to be significantly associated with C-reactive protein (CRP). CYP2C19 polymorphisms had no effect on voriconazole pharmacokinetic parameters. ‏Monte Carlo simulation based on CRP levels showed that a loading dose of 250 mg/12 h and a maintenance dose of 100 mg/12 h are recommended for patients with CRP ≤ 96 mg/L, whereas a loading dose of 200 mg/12 h and a maintenance dose of 75 mg/12 h are recommended for patients with CRP > 96 mg/L. The average probability of target attainment of the optimal dosage regimen in CRP ≤ 96 mg/L and CRP > 96 mg/L groups were 61.3% and 13.6% higher than with empirical medication, and the proportion of C_min_ > 5.5 mg/L decreased by 28.9%. In conclusion, the VRC PPK model for talaromycosis patients shows good robustness and predictive performance, which can provide a reference for the clinical individualization of VRC. Adjusting initial dosage regimens based on CRP may promote the rational use of VRC.

## Introduction

Talaromycosis is a fatal deep fungal infection caused by the biphasic fungus *Talaromyces marneffei* (*T. marneffei*), which is prevalent mainly in Southeast Asia and southern China (Vanittanakom et al., 2006; Hu et al., 2013). The disease was previously considered specific to individuals with HIV/AIDS; however, the number of non-HIV-related *T. marneffei* infections is rapidly increasing annually (Chan et al., 2016). In endemic areas, approximately 50,000 HIV-positive patients are newly infected with *T. marneffei* each year which results in up to 5,000 deaths annually (Hu et al., 2013; Ning et al., 2018). The mortality rate of talaromycosis can be as high as 91% with inadequate treatment, and antifungal therapy is the mainstay of treatment for this infection (Armstrong-James et al., 2014; Chan et al., 2016; Le et al., 2017; Jiang et al., 2019).

Voriconazole is a second-generation triazole with broad-spectrum antifungal activity recommended in international guideline for the treatment of talaromycosis (Panel on Antiretroviral Guidelines for Adults and Adolescents, 2019). Our previous retrospective studies showed that the clinical efficacy of VRC was similar to amphotericin B, and the incidence of adverse reactions was lower (Ouyang et al., 2017; Huang et al., 2021). However, the widely variation among VRC plasma concentrations were found during routine therapeutic drug monitoring (TDM) in these patients. Voriconazole exhibits nonlinear pharmacokinetic characteristics in adults with a large inter- and intra-patient variability of plasma concentrations (Hope, 2012; You et al., 2018). Those variants can partly be explained by age, body weight, CYP2C19 gene polymorphism, drug-drug interactions, liver diseases, and inflammation (Owusu Obeng et al., 2014; Veringa et al., 2017; Zhou et al., 2020). A large number of studies have indicated that the clinical efficacy and drug-related adverse reactions of VRC are closely related to its plasma concentrations (Miyakis et al., 2010; Troke et al., 2011; Dolton and McLachlan, 2014). In addition, the VRC target plasma trough concentration (C_min_) for patients with talaromycosis was reported to be >1 mg/L to ensure sufficient effect (2020), and supratherapeutic threshold of 6.0 mg/L was most predictive of toxicity (Luong et al., 2016). Voriconazole has a narrow therapeutic window, therefore, standardized dosage regimens are insufficient to achieve the targeted therapeutic exposure in different clinical settings (Chen et al., 2019). Although TDM can effectively improve VRC medication, however, unexpected adverse reactions and deviations from the therapeutic range may occur at baseline before TDM (Pascual et al., 2008; Park et al., 2012; Kim et al., 2019).

Population pharmacokinetics (PPK) provides a quantitative analysis of variables affecting pharmacokinetic parameters and is a common method in studies of individualized medicine. Although studies on VRC PPK have been widely reported, they mainly focused on critically ill patients or patients with liver dysfunction, hematological malignancies and organ transplant. Disseminated *T. marneffei* infection is a serious disease state that often causes multiorgan damage and the liver is one of the most commonly affected organs (Vanittanakom et al., 2006; Chan et al., 2016). In addition, the effects of hypoalbuminemia, inflammation, concomitant medications, and CYP2C19 gene polymorphisms complicate the clinical application of VRC.

The main purpose of this study was to evaluate the characteristics of VRC pharmacokinetics in patients with talaromycosis using nonlinear mixed effects modeling (NONMEM), in addition to determining the extent of interindividual variability in pharmacokinetics of VRC, and to provide dose recommendations based on the final model for promoting reasonable use of VRC.

## Materials and methods

### Patients

This prospective observational clinical study was conducted at The First Affiliated Hospital of Guangxi Medical University and People’s Hospital of Baise from February 2019 to November 2021. Patients aged ≥18 years with a confirmed diagnosis of talaromycosis treated with VRC as an initial treatment were included. The exclusion criteria were as follows: 1) pregnancy or lactation; 2) treatment with other antifungal agents or medications that could seriously affect the pharmacokinetics of VRC; 3) hepatic dysfunction with Child-Pugh score class C; 4) renal impairment with creatinine level more than three times the upper normal limit; 5) diagnosis of tuberculosis or undergoing chemotherapy. This study was approved by the Medical Ethics Committee of the First Affiliated Hospital of Guangxi Medical University (KS No. 2019008). The investigators conducted the study in accordance with good clinical practice guidelines, and all patients signed a written informed consent before participating in the study.

### Study medication and blood sampling

All patients received a standard dose of VRC according to the medication recommendations. The loading dose regimen was as follows: intravenous VRC 6 mg/kg q12 h on day 1 (or oral 400 mg q12 h on day 1) and followed by a maintenance dose of 4 mg/kg q12 h (or oral 200 mg q12 h) thereafter. The non-loading dose regimen was intravenous VRC 4 mg/kg q12 h or oral 200 mg q12 h. If the patient weighs less than 40 kg, the oral dose was reduced to half. A sparse sampling strategy was used to collect blood samples. Patients were randomly assigned to collect at least one blood sample within 30 min before administration and at 0.5, 1, 2, 4, 6, 8, 10, and 12 h after administration. The plasma concentration of VRC was considered to be at steady state after the 5th dose following the loading dose, or after 5 days without loading dose. Blood samples of C_min_ were collected within 30 min before the next dose under steady state. Blood samples were centrifuged within 6 h and stored at −80°C; plasma was used to detect the concentration of VRC, and white blood cells (WBCs) were used for CYP2C19 identification. VRC plasma concentrations were measured with automatic two-dimensional liquid chromatography (2D-HPLC, Demeter Instrument Co., Ltd., Hunan, China). The linearity range was 0.24–12.04 mg/L (*R* ^2^ = 0.9999) and the lower limit of quantitation was 0.2 mg/L. The assay precisions (intra-day and inter-day variability) were less than 1% (1.19–8.32 mg/L), and the online recovery was 91.38% ± 2.35% (Tang et al., 2019).

### Clinical data collection and CYP2C19 genotyping

Data were collected through a unified format from the hospital electronic medical record system; data included demographic information (sex, age, weight, height); underlying diseases (HIV); VRC medication information (date, dose, administration time, interval time); concomitant medications [proton pump inhibitors (PPIs), glucocorticoids]; and laboratory test results, including WBC, hemoglobin (HGB), platelet (PLT), neutrophil granulocyte (NEU), alanine aminotransferase (ALT), aspartate aminotransferase (AST), albumin (ALB), total protein (TP), total bilirubin (Tbil), glutamyl transpeptidase (GGT), urea (UREA), and C-reactive protein (CRP). CYP2C19 (*2/*3/*17) genotyping was performed by the Sanger dideoxy DNA sequencing method with an ABI3730xl-full automatic sequencing instrument (ABI Co.) from Qingke Biotechnology Co., Ltd., in Guangzhou. According to the CYP2C19 genotypes, the metabolic phenotype was classified into 5 categories: ultrarapid metabolizer (UM, CYP2C19 *17/*17), rapid metabolizer (RM, CYP2C19 *1/*17), extended metabolizer (EM, CYP2C19 *1/*1), intermediate metabolizer (IM, CYP2C19 *1/*2, CYP2C19 *1/*3, CYP2C19 *2/*17), and poor metabolizer (PM, CYP2C19 *2/*2, CYP2C19 *2/*3, CYP2C19 *3/*3).

### Population pharmacokinetic model

The VRC PPK model was constructed using nonlinear mixed-effects modeling software (NONMEM version 7.4.0 ICON Development Solutions, Ellicott City, MD). The data processing software included Perl-speaks-NONMEM (PsN, version 4.6.0) and Pirana (version 2.9.5a). Graphical analysis was performed using the *R* (version 4.1.3) and Xpose4 package (version 4.7.0). The first-order conditional estimation method with interaction (FOCE-I) was selected to estimate the model. One- and two-compartment models with first-order absorption and linear/non-linear elimination were compared to evaluate the best basic structural model. Population typical parameters such as clearance (CL), apparent volume of distribution (V) and oral absolute bioavailability (F) were estimated, and the absorption rate constant (Ka) was fixed at 1.1 h^−1^ as reported in the literature (Pascual et al., 2012). The inter-individual variability (IIV) in VRC PK parameters was described with an exponential model, which was expressed as follows:

$$P_{i}=\hat{P}\times e^{\eta^{i}}$$ (1)

where $P_{i}$ represents the ith individual parameter, $\hat{P}$ represents the typical value of the population parameter, and $\eta_{i}$ is the random effect of the ith individual, which is normally distributed, with a mean of zero and variance of $\omega^{2}$ . The residual variability (RSV) was tested with the following equations:

$$Proportional\;model\text{:}\;Y=F\times \left(1+\varepsilon_{1}\right)$$ (2)

$$Exponential\;models\text{:}\;Y=F\times e^{\varepsilon_{1}}$$ (3)

$$Additive\;model\text{:}\;Y=F+\varepsilon_{1}$$ (4)

$$Combined\;model\text{:}\;Y=F\times \left(1+\varepsilon_{1}\right)+\varepsilon_{2}$$ (5)

where Y is observed value, F is the model-predicted value, and $\varepsilon_{1}$ , $\varepsilon_{2}$ represents the residual variation in which the distribution obeys a normal distribution, with zero mean and variances of σ^2^.

Covariate model exploration was conducted after the selection of the basic model. The relationship between covariates and PK parameters was evaluated by plotting empirical Bayesian estimates against patient variables. The Stepwise method was adopted to screen covariates, and potential covariates were sequentially tested using forward inclusion to establish the full model, followed by a backward elimination procedure to obtain a final model. Covariates were centered by their medians and were explored with linear, proportion, power function, and exponential models. A covariate was considered to be significant when inclusion resulted in a decrease in the objective function value (OFV) > 3.84 (*p* < 0.05, χ^2^, df = 1) and an increase in the OFV > 6.63 (*p* < 0.01, χ2, df = 1) in the backward step. The optimal model meets the following criteria: 1) the OFV was minimized, 2) the goodness-of-fit (GOF) was improved, 3) the addition of covariates reduced the differences between individuals, 4) covariates were clinically reasonable, and 5) the 95% CIs for the parameter estimates did not include zero.

### Model validation

The goodness-of-fit plots were constructed to evaluate the adequacy of fitting, including individual prediction concentrations (IPRED) and population prediction concentrations (PRED) versus observed concentrations (DV), PRED and time after dose (TAD) versus conditional weighted residuals (CWRES), respectively. Bootstrapping was performed to assess the robustness and stability of the final model. One thousand resample datasets were generated from the original data, all parameters were re-estimated using the final model, and their medians and 95% confidence intervals (CIs) (2.5th percentile and 97.5th percentile) were calculated and compared with the final model parameters. Visual predictive checks (VPCs) were simulated 2,000 times to graphically assess the predicted performance of the final model. The 95% CIs for the 5th, 50th, and 95th percentiles of the simulated concentrations were calculated and compared with the observed concentrations.

### Dosage regimen simulations

Monte Carlo simulation was used to determine the initial optimal dosage regimens for VRC. Simulations of VRC trough concentrations under steady state conditions were conducted using the final model parameters. A total of 1,000 replicates of C_min_ were simulated for each dosage regimen. The target trough concentration range was defined as 1.0–5.5 mg/L (Hamada et al., 2012; Suzuki et al., 2013), and different loading and maintenance doses were simulated for each subpopulation stratified by the covariates included in the final model. A loading dosage regimen was considered appropriate if it achieved a higher probability of the C_min_ attaining the therapeutic range at 24 h (C24). Base on the optimal loading dose, the optimal maintenance dose regimen was considered to have a high probability of VRC steady-state trough concentration reaching the therapeutic range and a low probability of C_min_ > 5.5 mg/L. The IIV and RSV were included in this simulation.

### Statistical analysis

Linear regression analysis was used to analyze the correlation between significant covariates and clearance estimated by the final model, and Mann-Whitney *U* test was used to analyze the differences in CL distribution corresponding to different covariates. Statistical analysis was performed with SPSS software (version 24.0, IBM Corporation, Armonk, New York), and *p* < 0.05 was considered statistically significant.

## Results

### Patient characteristics

This study enrolled 69 patients diagnosed with talaromycosis from the First Affiliated Hospital of Guangxi Medical University and People’s Hospital of Baise. Specifically, 50 patients received IV loading dose with subsequent IV maintenance dose, 4 patients received oral loading and oral maintenance dose, 1 patient was IV route for loading dose with subsequent oral maintenance dose, and 14 patients were on non-loading dose (3 patients received intravenous and 11 patients received oral). A total of 233 VRC plasma concentrations (median of 4 per patient, range 1–9) including 75 C_min_ (median of 1 per patient, range 0–2) from 69 patients were obtained to analysis. Extensive inter-individual variability in C_min_ were observed in these patients, which ranging between 0.23 mg/L and 16.95 mg/L. Among the C_min_ values, 47.7% (31/65) VRC C_min_ values were maintained within 1.0–5.5 mg/L, while 12.3% (8/65) and 40.0% (26/65) of VRC C_min_ values were below 1.0 mg/L and above 5.5 mg/L, respectively. Table 1 summarizes the patients’ demographic information, laboratory test results and CYP2C19 genotypes. Among those patients, there were 8 poor metabolizers (PMs) (*2/*2, *2/*3), 31 intermediate metabolizers (IMs) (*1/*2, *1/*3), and 30 extended metabolizers (EMs) (*1/*1). No ultrarapid metabolizers (UM) and rapid metabolizer (RM) patients were found in the study group. Thirty four patients were HIV positive that diagnosed for the first time with no antiviral treatment history. The most common concomitant medications affecting the pharmacokinetics of VRC were PPIs (32/69), and the maximum daily dose not exceed 40 mg.

**TABLE 1:** Patient demographic characteristics and laboratory data.

	The first affiliated hospital of Guangxi medical university (*n* = 35)		People’s hospital of Baise (*n* = 34)	
Characteristic	Mean ± SD	Median, range	Mean ± SD	(Median, range)
Male [*n* (%)]	24 (68.6)	—	31 (91.2)	—
Age (years)	58.1 ± 4.62	57 (54∼69)	38.0 ± 10.4	30 (20∼65)
WT (kg)	62.7 ± 6.66	61 (52∼72)	52 ± 9.65	50 (38∼87)
Laboratory				
ALB (g/L)	26.9 ± 4.02	26 (17.4∼36.0)	25.7 ± 5.9	24.7 (1∼43.8)
ALT (U/L)	26.1 ± 14.6	27.2 (4.0∼61.0)	46.0 ± 44.7	29 (10.0∼236)
CRP (mg/L)	93.9 ± 71.0	70.5 (1.6∼207.7)	61.3 ± 41.8	59.1 (0.9∼202)
DBIL (μmol/L)	4.76 ± 4.29	3.3 (1.2∼16.7)	10.5 ± 12.8	5.2 (1.0∼102.2)
GGT (U/L)	260.9 ± 176.2	290.1 (19.0∼662.0)	153.5 ± 174.8	95 (20∼1154)
PLT (10^9^/L)	437.4 ± 113.2	417 (246.1∼625.7)	123.7 ± 88.8	102 (6∼451)
TP (g/L)	69.2 ± 12.2	65.4 (54.7∼95.4)	61.8 ± 11.9	61.1 (43∼103.2)
WBC (10^9^/L)	15.16 ± 3.71	15.7 (7.04∼27.81)	3.79 ± 2.41	3.6 (1.0∼16.2)
UREA (mmol/L)	6.42 ± 2.55	5.81 (2.5∼10.97)	4.83 ± 3.25	4.0 (0.95∼18.47)
CYP2C19 phenotype^a^				
EM [*n* (%)]	13 (37.1)	—	17 (50)	—
IM [*n* (%)]	18 (51.4)	—	13 (38.2)	—
PM [*n* (%)]	4 (11.4)	—	4 (11.8)	—
Complications				
HIV infection [*n* (%)]	0	—	34 (100%)	—
Concomitant				
Omeprazole [*n* (%)]	4 (11.4)	—	10 (29.4)	—
Pantoprazole [*n* (%)]	2 (5.7)	—	1 (2.94)	—
Lansoprazole [*n* (%)]	6 (17.1)	—	0	—
Rabeprazole [*n* (%)]	2 (5.7)	—	0	—
Glucocorticoid [*n* (%)]	6 (17.1)	—	1 (2.94)	—

ALB, albumin; ALT, alanine aminotransferase; CRP, C-reactive protein; DBIL, direct bilirubin; GGT, glutamyl transpeptidase; HGB, hemoglobin; PLT, platelet count; TP, the total protein; UREA, urea nitrogen; WBC, white blood cell count; WT, weight; EM, extensive metabolizers; IM, intermediate metabolizer; PM, poor metabolizer.

a All gene frequencies fit Hardy-Weinberg genetic equilibrium (*p* > 0.05).

### Population pharmacokinetics model development

A one-compartment model with first-order absorption and elimination adequately described the data. Compared with the linear one-compartment model, neither the two-compartment model nor the nonlinear Michaelis-Menten elimination model can significantly reduce the OFV and improve the fitting effect. Individual variability in PK parameters can be well fitted by an exponential error model, and a combined model was used to describe the residual variability. The stepwise covariate modeling procedure showed that CL was significantly affected by CRP (ΔOFV = 8.302). EM status (ΔOFV = 5.235) on V was found to be significant during only forward selection. The population parameter estimates for the final model are presented in Table 2 and the equations of the final model are as follows. Typical values of CL, V, F were 4.34 L/h, 97.4 L, and 95.1%, respectively.

$$CL\;\left(L/h\right)=4.34\times e^{-0.135\times \left(\frac{CRP\left(mg/L\right)}{43.6}\right)}\times e^{1.01}$$ (6)

$$V\left(L\right)=97.4\times e^{0.0973}$$ (7)

$$Ka\left(h^{-1}\right)=1.1^{h-1}\left(Fixed\right)$$ (8)

F=95.1% (9)

**TABLE 2:** Parameters estimates of basic, final model and Bootstrap results.

	Basic model	Final model	Bootstrap	
	Estimate (RSE %)	Estimate (RSE %)	Median	2.5%–97.5% CI
OFV	300.176	291.874	271.66	200.39–343.32
PK parameters				
CL (L/h)	3.43 (13.8)	4.34 (18.6%)	4.39	2.86–4.46
CRP on CL	—	−0.135 (65.1%)	−0.151	−0.367–0.098
V (L)	95.2 (8.2%)	97.4 (7.1%)	97.3	84.5–111.9
k_a_ (h^−1^)	1.1 (Fixed)	1.1	1.1	1.1
F_1_ (%)	81.8 (25.7%)	95.1 (20.5%)	93.7	46.4–134
Inter-individual variability				
IIV_CL (%)	101.5 (11.4) (6.65%)^a^	100.5 (11.7%) (6.50%)^a^	101.2	77.4–124.1
IIV_V (%)	32.9 (16.3%) (41.5%)^a^	31.2 (14.7%) (42.5%)^a^	31.4	18.4–43.1
Residual-variability				
RSV_CV (%)	7.0 (18.4%) (19.9%)^a^	7.1 (16.5%) (19.8%)^a^	7.42	4.15–9.50
RSV_SD (mg/L)	0.387 (16.8%) (19.9%)^a^	0.373 (13.5%) (19.8%)^a^	0.109	0.0313–0.203

a Represents the shrinkage value of inter-individual variation and residual variation.

CL, clearance; V, distribution volume; CRP, c-reactive protein; CV, coefficient of variation; IIV, inter individual variability; RSV_CV, proportional residual variation; RSV_SD, additive type residual variation.

### Model evaluation

**FIGURE 1:**
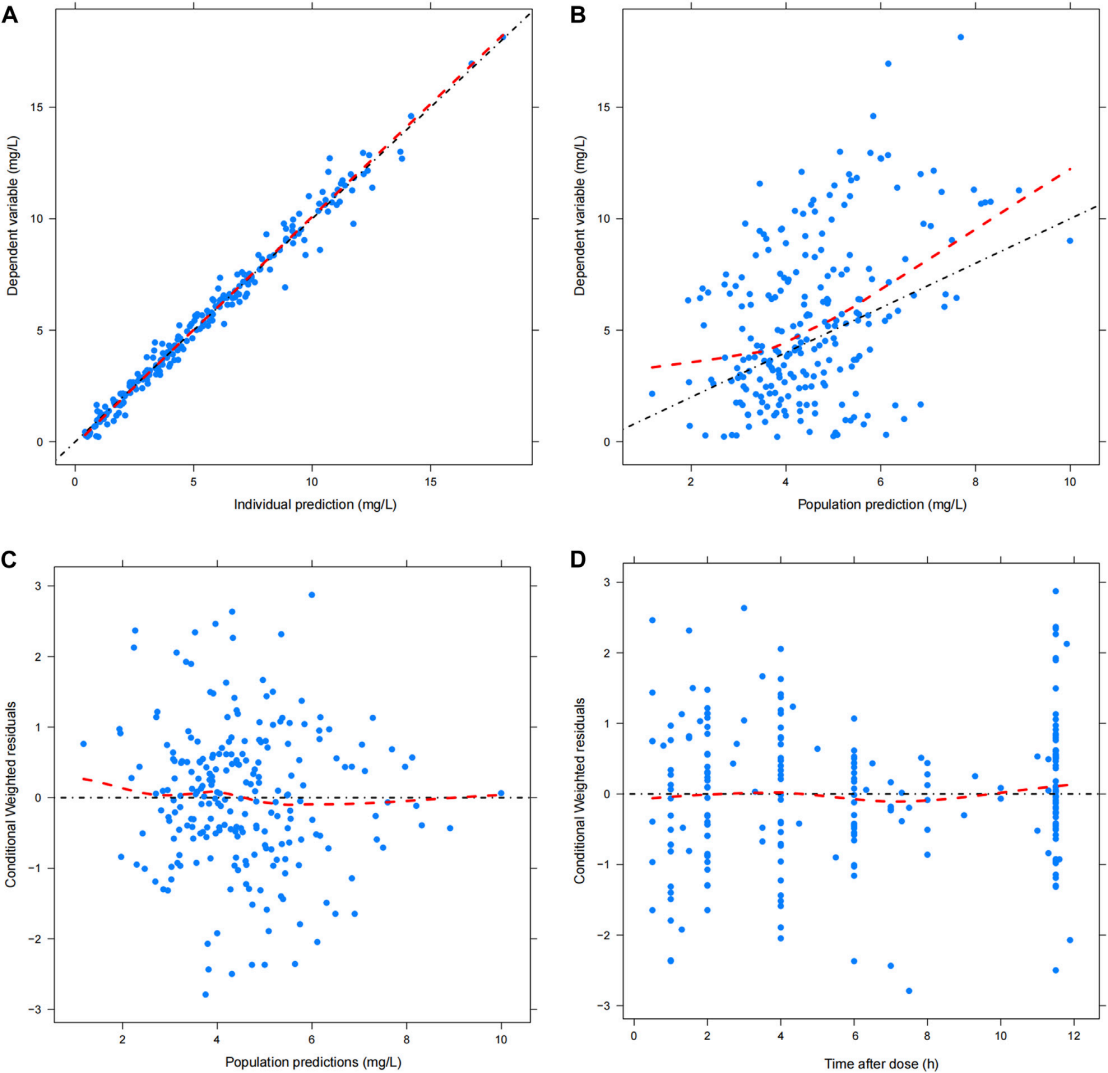
Goodness of fit plot of final model. **(A)** Scatter plot of observed value and individual predicted value; **(B)** Scatter diagram of observed value and population predicted values; **(C)** Scatter plot of conditional weighted residuals and predicted population value; **(D)** Scatter plot of conditional weighted residuals and time after dose. The black dotted line is the reference line, and the red dotted line is the LOESS trend line.

**FIGURE 2:**
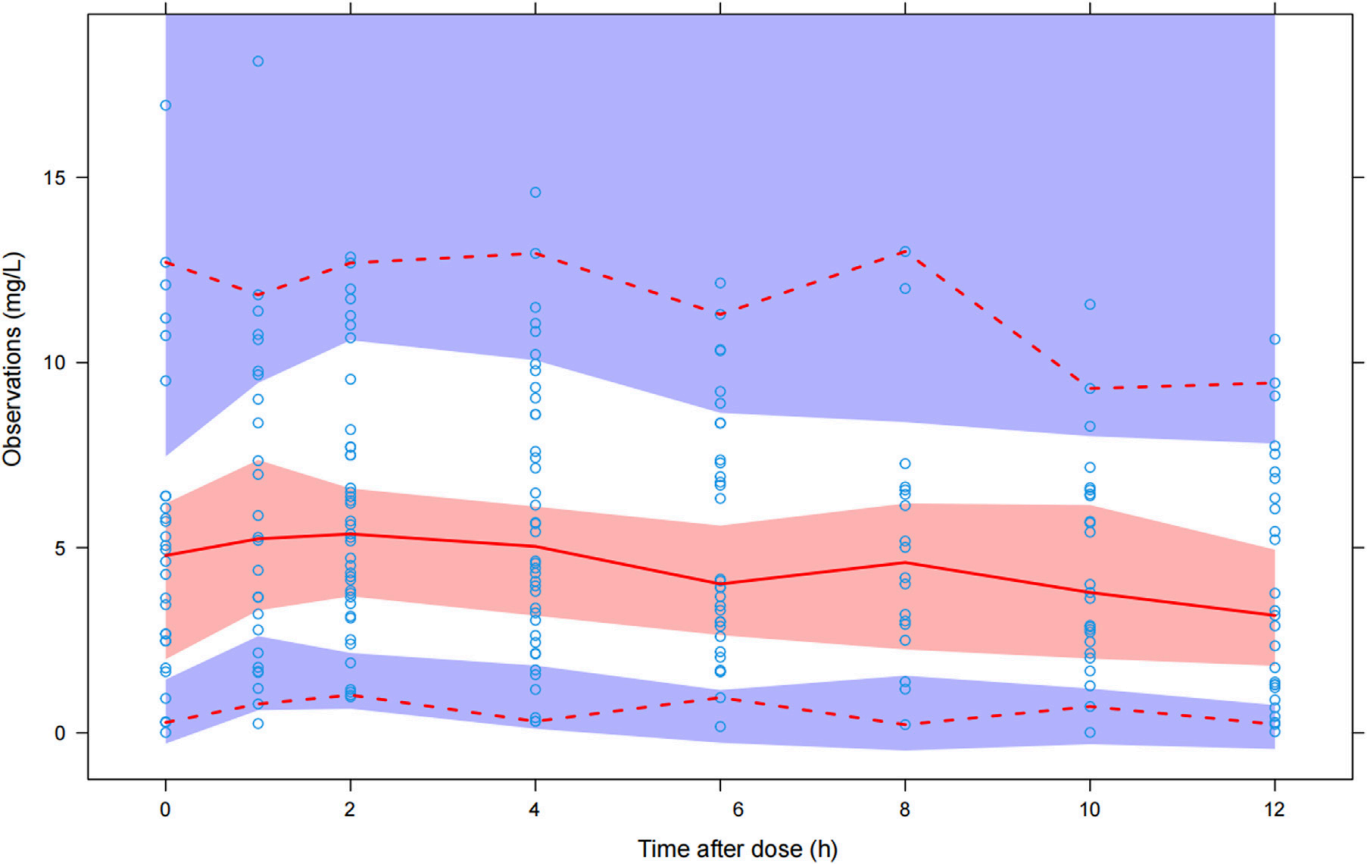
Visual predictive check of the final model. The blue hollow dots represent the original observed date of VRC blood drug concentration, the solid red line represents the median observed plasma concentration, and the red area represents the 95% confidence interval of the simulation-based median. The red dotted line represents the 5% and 95% percentiles of observed plasma concentration, the area formed between the two red dotted lines is called the 90% prediction interval (PI), and the blue area is the 95% confidence interval for the corresponding percentile predicted by the model.

The GOF chart of the final model illustrated that both the individual predicted value (IPRED) and predictive value (PRED) corresponded well with the observed value (DV). The conditional weighted residuals (CWRES) versus time and PRED showed good scattering, with most points located between ±2 (Figure 1). The bootstrap results demonstrated strong stability of the final model, with 986 out of 1,000 bootstraps being successful. Furthermore, the bootstrap estimates were similar to the typical values of the final model, and the 95% CI completely overlapped with the final model parameter (Table 2). The VPC visual verification diagram of the final model (Figure 2) shows that the majority of VRC DVs were covered in the 90% prediction interval (PI), indicating that the prediction results of the model were credible, with high predictive accuracy and good stability.

### Dosage regimen simulations

Monte Carlo simulations were utilized to determine the optimal dosage regimens for patients with *T. marneffei* infection based on the final model that included the significant covariate of CRP for calculating CL.

**FIGURE 3:**
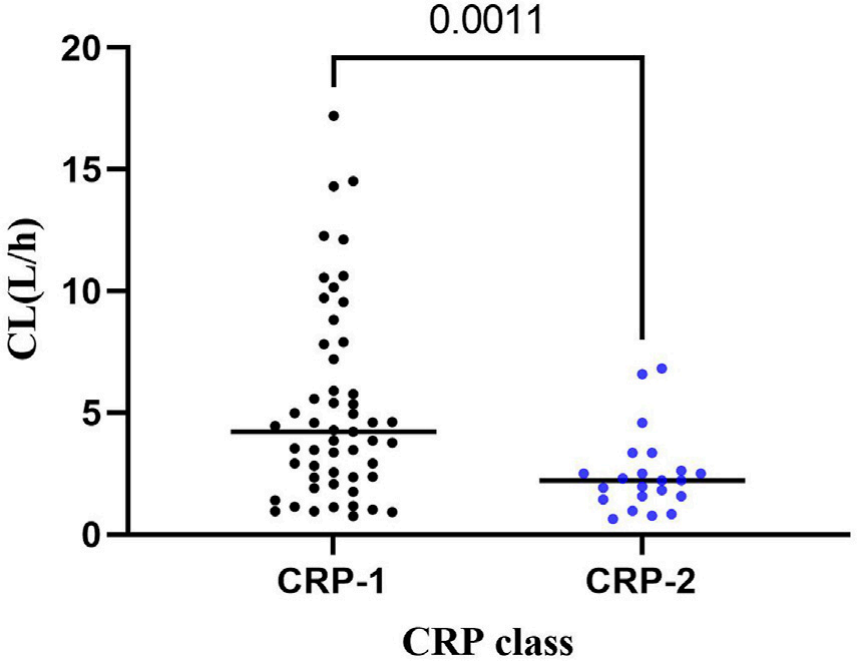
Distribution of VRC CL in CRP-1 and CRP-2 groups. CRP-1: CRP ≤ 96 mg/L; CRP-2: CRP > 96 mg/L.

Linear regression analyses were used to analyze the correlation between VRC CL at each trough concentration and the CRP levels obtained within the same period for patients who measured multiple trough concentrations on different occasions. A significant correlation was observed between VRC CL and CRP (*p* < 0.05). The CL values were also analyzed by stratifying the CRP and the VRC CL, and a significant difference (*p* < 0.001) between the two groups was observed: CRP ≤ 96 mg/L (CRP-1 group) and CRP > 96 mg/L (CRP-2), and CRP-2 group had a significantly lower CL than CRP-1 group (median: 2.23 vs. 4.23 L/h, Figure 3).

**FIGURE 4:**
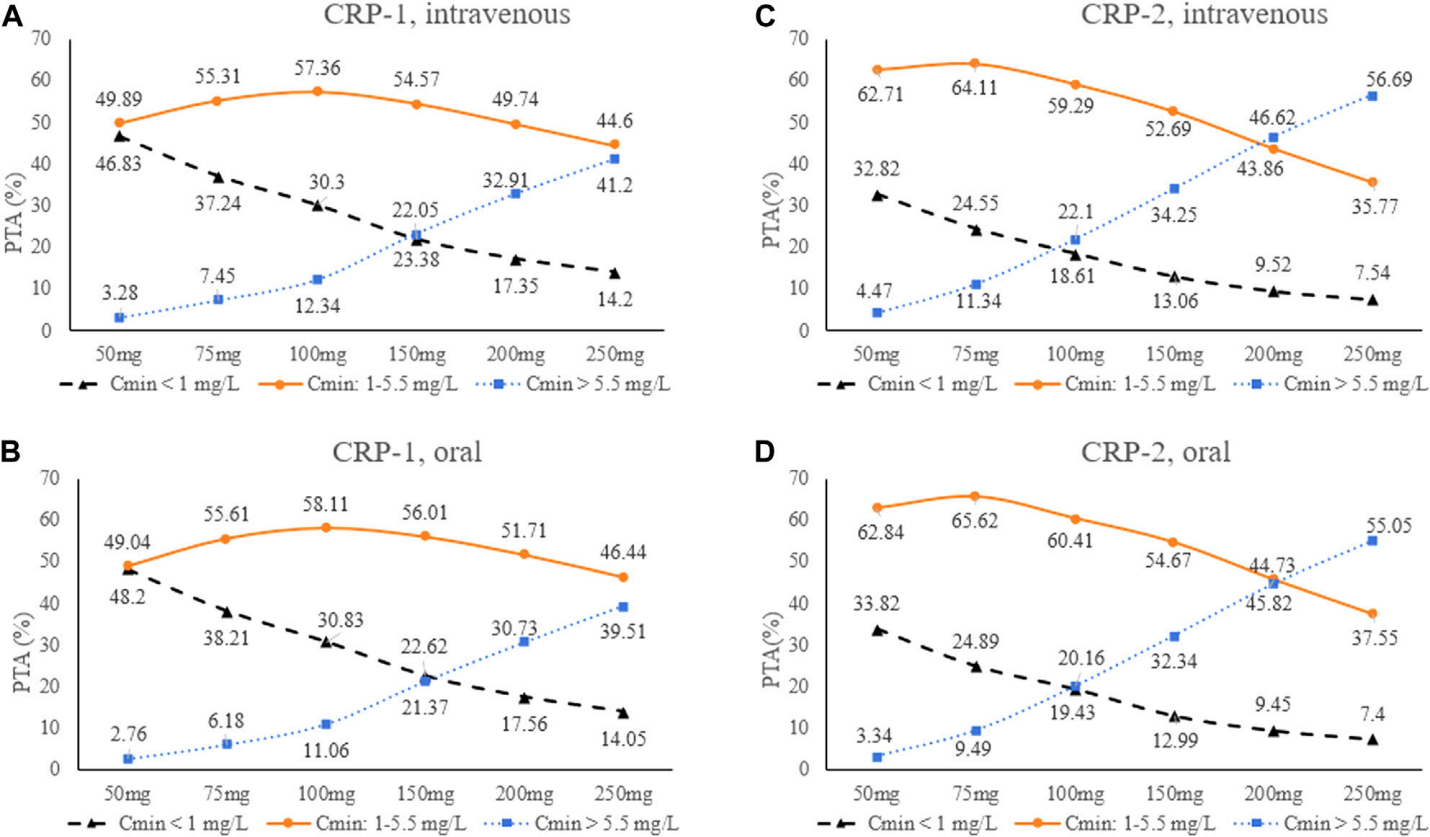
Trough concentrations distribution at different VRC maintenance doses for the four scenarios. **(A)**: CRP-1, intravenous; **(B)**: CRP-2, intravenous; **(C)** CRP-1, oral; **(D)**: CRP-2, oral. CRP-1:CRP ≤ 96 mg/L; CRP-2: CRP > 96 mg/L.

Therefore, the population was divided into two groups (CRP-1 and CRP-2) for Monte Carlo simulation. The results of intravenous (1 h infusion) and oral loading doses (200, 250, 275, 300, 350 mg q12 h) were shown in Table 3. According to the results, a loading dose of 250 mg/12 h was adequate for CRP-1 group patients to obtain high probabilities (intravenous: 80.17%; oral: 81.17) of the C_24_ attaining the VRC therapeutic range (1.0–5.5 mg/L). For CRP-2 group patients, 200 mg/12 h was more appropriate, and the probability of target attainment (PTA) of intravenous and oral was 86.45% and 86.79%, respectively. Based on the optimizing loading dose, various intravenous (1 h infusion) and oral maintenance dose regimens were simulated for the two groups (Figure 4). The final recommended regimens are shown in Table 4. For patients in the CRP-1 group, a loading dose of 250 mg/12 h and a maintenance dose of 100 mg/12 h were recommended, while for patients in the CRP-2 group, a loading dose of 200 mg/12 h and a maintenance dose of 75 mg/12 h were recommended. The overall average PTA of the optimal administration regimen is 61.3%, 13.6% higher than that of experiential administration. Supratherapeutic concentration (C_min_ > 5.5 mg/L) decreased by 28.9%.

**TABLE 3:** Probability of C_24_ reaching the treatment range in two groups with different voriconazole loading doses on the first day.

Loading dose (for the first 24 h)	PTA (%)					
	CRP-1			CRP-2		
	<1 mg/L	1–5 mg/L	>5 mg/L	<1 mg/L	1–5 mg/L	>5 mg/L
200 mg/12 h, i.v.	20.45	78.61	0.94	11.73	86.45	1.82
200 mg/12 h, po	21	78.39	0.61	11.89	86.79	1.32
250 mg/12 h, i.v.	16.96	80.17	2.87	8.54	84.38	7.08
250 mg/12 h, po	15.73	81.17	3.1	8.81	85.57	5.62
275 mg/12 h, i.v.	14.33	79.17	6.5	7.56	81.29	11.15
275 mg/12 h, po	14.14	80.49	5.37	7.42	83.36	9.22
300 mg/12 h, i.v.	12.81	77.08	10.11	6.8	76.75	16.45
300 mg/12 h, po	13.14	78.59	8.27	6.76	79.77	13.47
350 mg/12 h, i.v.	11.11	70.4	18.49	5.64	66.51	27.85
350 mg/12 h, po.	11.17	72.65	16.18	5.4	70.39	24.21

iv: Intravenous; po: Oral; CRP-1: CRP ≤ 96 mg/L; CRP-2: CRP > 96 mg/L; the black outer box indicates the scheme with the highest PTA.

**TABLE 4:** Recommended initial dosing regimen of voriconazole for patients in the two groups.

Condition	Recommendation	PTA (%)			
		<0.5 mg/L	1.0∼5.5 mg/L	>5.5 mg/L	*C*_ss, trough_ (mg/L)
CRP-1	LD: 250 mg/12 h; iv	30.3	57.36	12.34	2.61
	MD: 100 mg/12 h, iv				
	LD: 250 mg/12 h; po	30.83	58.11	11.06	2.52
	MD: 100 mg/12 h, po				
CRP-2	LD: 200 mg/12 h; iv	24.55	64.11	11.34	2.73
	MD: 75 mg/12 h, iv				
	LD: 200 mg/12 h; iv	24.89	65.62	9.49	2.62
	MD: 75 mg/12 h, iv				

LD, loading dose; MD, maintenance dose; iv, Intravenous; po, Oral; CRP-1, CRP ≤ 96 mg/L; CRP-2, CRP > 96 mg/L.

## Discussion

This prospective study developed a population pharmacokinetics model of VRC in patients with talaromycosis. In this study, VRC plasma C_min_ demonstrate high interindividual variability with 52.3% of C_min_ outside the therapeutic range, which indicate the need to adjust VRC dose in these patients to optimize VRC plasma concentrations. Interestingly, CRP was identified as a promising biomarker for optimizing the initial dose of VRC rather than CYP2C19 genotype.

A one-compartment linear elimination model was used to describe the pharmacokinetic characteristics of VRC in this study, which is consistent with previously published PPK model in adult patients (Shi et al., 2019). The typical population values for CL, V, and F of VRC in this study were 4.34 L/h, 97.4 L and 95.1%, respectively. The Ka was fixed at 1.1 h^−1^ due to the limited number of samples in the absorption phase. (Nomura et al., 2008; Han et al., 2010; Han et al., 2011; Pascual et al., 2012; Wang et al., 2014; Chen et al., 2015b; Li et al., 2017; Lin et al., 2018).The population estimates of CL in this study were similar to the values in patients with hematologic malignancies (Liu et al., 2019) and pulmonary disease (Chen et al., 2015a), and were higher than liver dysfunction patients (Ren et al., 2019; Tang et al., 2021; Wang et al., 2021), and were lower than those in patients with kidney transplant (Li et al., 2017), hematopoietic stem cell transplant patients (Chen et al., 2019) and healthy volunteers (Purkins et al., 2002). The reason for the increase in VRC plasma concentrations may be related to pathological damage to hepatocytes caused by aggressive infection of *T. marneffei*, which results in VRCs with lower clearance rates than other populations. The IIV of CL (100.5%) was high compared with the median (range) values obtained from other studies [41% (21.3%–107%)], showing large interindividual variation. While the IIV of V (31.2%) is similar to other studies [32.75% (12%–98%)]. The RSV was 7.1% and quite low compared with that of other studies, which ranged from 13% to 61% (Nomura et al., 2008; Han et al., 2010; Han et al., 2011; Pascual et al., 2012; Wang et al., 2014; Chen et al., 2015b; Li et al., 2017; Lin et al., 2018). The shrinkage of CL and V are 6.5% and 42.5%, respectively. The shrinkage of the V is a little high probably due to insufficiency of the data in the absorption phase.

The study identified CRP was the most significant covariate affecting the pharmacokinetic parameters of VRC, which are consistent with the results observed by Alffenaar JW et al. (van Wanrooy et al., 2014; Encalada Ventura et al., 2015; Veringa et al., 2017). C-reactive protein is an acute protein mainly produced by hepatocytes that increase dramatically in response to injury, infection, and inflammation, which plasma level can reflect the severity of inflammation (Sproston and Ashworth, 2018). Invasive *T. marneffei* infection can cause multiple organ damage and CRP is significantly increased in patients with poor prognosis (Sun et al., 2021; Shi et al., 2022). Studies have shown that drug-metabolizing enzymes, including cytochrome P450 (CYP) isoenzymes, are decreased at the transcriptional levels during infection or inflammation, which leading to reduce the metabolism of VRC (Renton, 2004; Aitken and Morgan, 2007; van Wanrooy et al., 2014; Encalada Ventura et al., 2015; Veringa et al., 2017). Therefore, the influence of inflammation needs to be considered when deciding the dose of VRC to avoid over-dosing administration.

Based on the final model, intravenous and oral loading dose of 250 mg q12 h and 200 mg q12 h for patients in CRP-1 and CRP-2 group were sufficient to achieve the C_min_ target at 24 h with high average probability of 80.67% and 86.62%, respectively. These results indicate that high CRP level can increase VRC exposure, and patients with severe inflammation received lower doses than conventional clinical medication, and that the percentage of VRC C_min_ > 5.5 mg/L increased significantly with increasing dose. Furthermore, the simulation results of this study found that PTA obtained by intravenous and oral routes were very close under the same conditions (Table 3; Figure 4). It may indicate that oral and intravenous administration could be alternated depending on the patient’s condition. In brief, the recommended medication regimen improved the effectiveness and safety of VRC treatment, which can provide a reference for clinical application.

Hypoalbuminemia is also one of significant characteristics in these patients, which likely to have been caused by an imbalance between ALB synthesis and catabolic rate, and secondary to an active inflammation (Chantharit et al., 2020). Hypoalbuminemia increases VRC plasma unbound drug concentration which may lead to drug-related adverse reactions (Khan-Asa et al., 2020; Yuan et al., 2020; Takahashi et al., 2021). In this study, hypoalbuminemia did not show to be a significant covariate of VRC plasma exposure. Albumin showed a correlation with the interindividual variation of CL, but was eventually excluded as its OFV decrease in the process of forward inclusion was less than 3.84, which might be related to the baseline value of ALB and the lack of significant improvement after treatment in the short term.

Voriconazole is metabolized mainly by CYP2C19 enzymes in the liver, and CYP2C19 polymorphisms are thought to significantly affect VRC metabolism (Lamoureux et al., 2016; Clinical Pharmacogenetics Implementation Consortium, 2018). Previous studies have shown that the proportion of poor metabolizers was significantly higher in the Asian population than in the Caucasian population, and poor metabolism resulted in an increase in plasma VRC concentration (Mizutani, 2003). In our study, the CYP2C19 genotype was determined in all patients, and the gene frequency distribution was similar to previous studies in Asia and did not deviate from Hardy-Weinberg Equilibrium (Mizutani, 2003; Jose et al., 2005; Vu et al., 2019). Nevertheless, the results of our analysis indicate that there was no significant correlation between CYP2C19 phenotype and VRC plasma concentration. This result needs further investigation in future studies with larger sample size.

Drug-drug interactions (DDIs) in VRC are also one of the concerns of clinicians, as they can cause changes in VRC plasma concentrations. Common concomitant medications include PPIs and glucocorticoids, although the relationship between these drugs and VRC remains inconclusive (Hoenigl et al., 2013; Niece et al., 2015). Proton pump inhibitors are widely used drugs and also undergo CYP450-dependent metabolism, thereby competitively inhibiting the metabolism of VRC (Niece et al., 2015). Voriconazole plasma exposure may be related to the type and dosage of PPIs, and omeprazole has the strongest inhibitory effect on VRC metabolism (Qi et al., 2017; Blanco Dorado et al., 2020). In this study, no significant effect of concomitant medication (PPIs) on VRC plasma concentration was detected, which may be related to the low frequency of omeprazole use. Additionally, DDIs between VRC and immunosuppressants were not evaluated in HIV-infected patients because antiretroviral treatment was not initiated during voriconazole induction therapy.

Our study has some notable limitations. One is that there might be some deviation in the results due to the small sample size. In addition, the variables included in this study were limited and cannot fully explain the factors influencing individual pharmacokinetic variation of VRC. In conclusion, a population pharmacokinetic model for VRC in patients with talaromycosis was developed, providing valuable information for individualized use of VRC and further assistance in guiding the treatment of patients with talaromycosis.

## Data Availability

The data analyzed in this study is subject to the following licenses/restrictions: The data are not publicly available due to privacy or ethical restrictions. Requests to access these datasets should be directed to Taotao Liu, liutaotao@gxmu.edu.cn.
